# Optimising prostate cancer pathways: Improving post‐biopsy waiting times in a tertiary centre

**DOI:** 10.1002/bco2.70045

**Published:** 2025-07-02

**Authors:** Shayan Soroush, Sean Lim, Prachi Beniwal, Gavin Wei, Ying Lu, Kylie Yen‐Yi Lim, Kirsten Holden, Matt Harper, Scott Donnellan, Weranja Ranasinghe

**Affiliations:** ^1^ Faculty of Medicine, Nursing & Health Sciences Monash University Australia; ^2^ Department of Urology Monash Health Australia; ^3^ Department of Surgery Monash University Australia

**Keywords:** cancer care pathway, multidisciplinary team, patient satisfaction, prostate biopsy, prostate cancer, waiting times

## Abstract

**Objectives:**

To evaluate the effectiveness of a streamlined Prostate Cancer Care Pathway (PCCP) in reducing post‐biopsy waiting times and improving patient satisfaction in a high‐volume tertiary centre.

**Patients and Methods:**

Patients undergoing prostate biopsies were prospectively followed through PCCP for one year and were retrospectively compared to 150 patients who were treated at our centre either immediately prior to PCCP implementation (2022) or during Covid‐19 lockdowns (2020). Waiting times were compared using the Kruskal‐Wallis H‐test. Patient satisfaction was assessed using the modified PCa Questionnaire for Patients (PCQ‐P).

**Results:**

A total of 398 patients were included. 248 patients went through PCCP, compared with 75 patients pre‐PCCP implementation (2022) and 75 patients during the 2020 pandemic. The median time from biopsy to results post‐PCCP was 15.0 days (IQR 13.0–19.0). This was significantly shorter than pre‐PCCP introduction of 21.0 days (17.0–28.0) and during 2020 lockdowns, 18.0 days (14.0–21.0, p < 0.001). A total of 131 patients (52.8%) requiring treatment under PCCP were streamlined for multidisciplinary discussion following imaging at a median time of 38.0 (29.8–42.0) days and seen at Urology or Radiation Oncology Consultant clinic for treatment discussion at a median of 38.0 days (31.0–49.0), compared to 63.0 days (45.0–84.0) pre‐PCCP (2022) and 52.0 days in 2020 (38.0–75.0, p < 0.001). A total of 176 PCCP patients (70.1%) participated in PCQ‐P with 93.2% of participants reporting satisfaction with waiting time durations (n = 176).

**Conclusion:**

PCCP implementation reduced waiting times in all post‐biopsy care measures following significant Covid‐19 delays in PCa care delivery. Streamlining resources using similar pathways can reduce waiting times in cancer care and other conditions to alleviate anxiety during healthcare system strain.

## 1 INTRODUCTION

Prostate Cancer (PCa) is the fourth‐most commonly diagnosed cancer worldwide, responsible for over 396 000 annual deaths globally.^1^ PCa diagnostic and treatment delays are well‐recognised challenges to Urology departments, particularly amongst patients with higher‐risk disease who may require more time for staging and complex therapeutic discussions^2^, ^3^, ^4^ Cancer care pathways (CCPs) have been increasingly adopted to streamline diagnostic and treatment processes for various cancers with their aims to improve care planning, multi‐disciplinary team (MDT) communication and patient satisfaction.^5^ External stressors including the Covid‐19 pandemic have highlighted vulnerabilities within healthcare systems, compounding delays and heightening patient anxiety in prostate cancer management.^6^, ^7^ These challenges highlight the need for innovative approaches to ensure consistent, patient‐centred care delivery, even in times of systemic strain.

Despite the proliferation of CCPs, substantial gaps persist in their overall effect on cancer care, particularly regarding waiting times to receive results and discuss treatment following biopsy. For instance, Valbekmo et al. highlighted that while centralisation of PCa care through CCPs can enhance care quality, it may inadvertently extend waiting times as a result of extra investigative or management steps associated with these pathways; however, their study did not quantify these delays or patient satisfaction.^3^ The Australian CCP endorsed by the Cancer Council provides recommended intervention within 3 months of diagnosis for PCa, or within 4 weeks for patients with symptoms.^8^ Similarly, the National Health Service (United Kingdom) CCP guide for PCa care has 14‐day, 21‐day and 28‐day best practise timed pathways to reduce anxiety and variation in waiting times whilst awaiting prostate biopsy results.^9^ Although these structured CCPs and national benchmarks exist, there is a notable paucity of research evaluating their local implementation at high‐volume tertiary centres as well as their effects on actual waiting times and patient satisfaction.

This study evaluates the implementation of a novel Prostate Cancer Care Pathway (PCCP) in a high‐volume tertiary academic centre. The PCCP is designed to minimise post‐biopsy waiting times for disclosing biopsy results, MDT meetings and discussing treatment options with patients, as well as improving patient satisfaction. By optimising existing resources and integrating multidisciplinary collaboration, the PCCP represents a pragmatic approach to mitigating delays in PCa care. Our findings contribute to the broader discourse on efficient healthcare delivery models, offering actionable insights into alleviating patient anxiety and enhancing care outcomes in prostate cancer management.

## 2 PATIENTS AND METHODS

### 2.1 Study design and outcome measures

This prospective cohort study was conducted at Monash Health (Victoria, Australia) and approved by the Monash Health research office (HREC reference: QA/88183/MonH‐2022‐325 157[v1]). All patients who underwent prostate biopsy and subsequently followed the PCCP from the date of its implementation on 08/07/2022 for a duration of one year until 05/07/2023 were included.

The PCCP is a risk‐stratification model developed by the Urology Department at Monash Health to optimise prostate cancer (PCa) care by guiding ideal timelines for key steps following prostate biopsy. These include the initial appointment where biopsy results are disclosed to patients, MDT meetings to consider individualised treatment recommendations and discussing treatment options with patients as required. The primary outcome measure was the intervals between these steps, which represent the waiting times experienced by patients.

Patients undergoing the PCCP were compared with two previous cohorts to enable comparison of waiting times. The first control cohort was 75 patients who had their initial post‐biopsy appointment immediately prior to PCCP implementation, between 18/02/2022 and 06/07/2022. The second control cohort was 75 patients who had their initial post‐biopsy appointment during the second Covid‐19 lockdown in Melbourne, Victoria between 09/07/2020 and 27/10/2020 to assess the impact of the Covid‐19 lockdown on patients' waiting times. Patients were excluded if they had insufficient follow‐up data or pursued follow‐up and/or treatment outside of Monash Health. Additionally, patients with previous prostate biopsies who were on active surveillance (AS) or watchful waiting were not excluded.

For patients that underwent the PCCP, the secondary outcome was patient satisfaction regarding the pathway using the PCa Questionnaire for Patients (PCQ‐P),^10^ which has acceptable validity and reliability in hospital clinic settings.^11^ The questionnaire was modified with selected questions from Sections C and D, which pertain to diagnosis and treatment that are specific to the PCCP (Supplementary Material). All PCCP patients were offered opportunities to participate in the phone questionnaire as part of clinic follow‐up. The questionnaire was not performed on patients prior to PCCP implementation due to the high possibility of recall bias.

The PCQ‐P consisted of two parts. The first applied to all PCCP patients, including questions relating to waiting times for Urology appointments and waiting times for receiving biopsy results. The second part only applied to patients with PCa diagnoses and included questions relating to time provided to decide on treatments, and waiting times for treatment commencement. Patients were only included in Part 2 of the questionnaire if they had discussed their treatment plan with a Urology and/or Radiation Oncology Consultant.

### 2.2 Patient pathways

**FIGURE 1:**
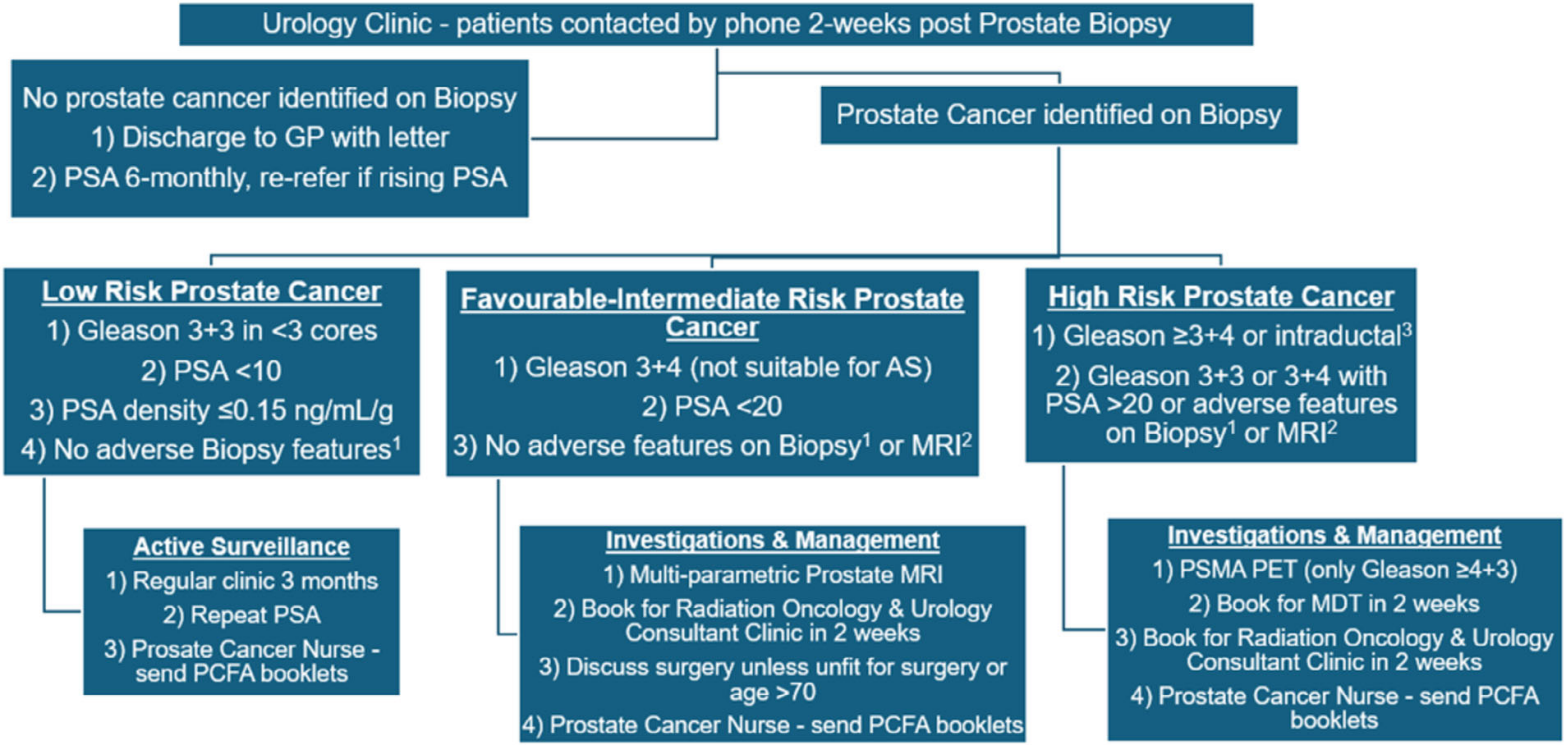
PCCP pathways.

Based on grading, MDT discussion and Radiation Oncology/Urology review, patients were stratified into various pathways of PCa care depicted in Figure 1.

Patients with benign biopsies were discharged to their GP with clinic letters and repeat PSA in 6 months. Patients with a new diagnosis of low‐risk PCa (Gleason 3 + 3 in less than three cores, pre‐operative PSA < 10 ng/ml and no adverse features such as intraductal/ductal carcinoma or BRCA mutation family history) were placed on AS.

Patients with a new diagnosis of favourable‐intermediate risk PCa (Gleason 3 + 4, pre‐operative serum PSA < 20 ng/ml and no adverse features on biopsy (including intraductal/ductal cancer or BRCA mutation family history) or MRI adverse features (including lymph node involvement, extracapsular extension or seminal vesicle involvement)) were referred to discuss treatment with a Radiation Oncologist and Urology clinic at two weeks. The PCa Nurse would also contact these patients to share information including PCa Foundation of Australia (PCFA) booklets to guide newly diagnosed patients^12^ and provide additional day‐to‐day support.

Patients with a new diagnosis of unfavourable‐intermediate risk PCa (Gleason 4 + 3) and high‐risk PCa (Gleason >4 + 3) were streamlined to undergo PSMA PET scans and were booked for MDT discussion within two weeks. They were also referred to a same‐day Radiation Oncology and Urology Consultant Clinic to discuss treatment at two weeks.

### 2.3 Data collection

Local clinic records were used to retrieve lists of patients from the three aforementioned cohorts. Patients' age, pre‐operative serum PSA level, pathological biopsy characteristics (Gleason scores and ISUP categories), MDT meeting dates and the dates of all subsequent Urology and/or Radiation Oncology follow‐up appointments until the first appointment where treatment discussions took place were noted from electronic medical records. Gleason grade group was included in five categories 1, 2, 3, 4 and 5. Data was collected from medical records in a standardised, secure access platform by four data collectors (SS, PB, SL, YL).

The PCQ‐P was performed by the same operator (SS) for all participants, who contacted patients through a private number and read the scripted introduction, questions and answer options. Patients were contacted at least three months following their initial post‐biopsy review clinic appointment. Patients were excluded from the questionnaire if they refused participation or if they did not answer the phone on two separate occasions. Interpreter services were made available for patients with language barriers.

### 2.4 Statistical analysis

Descriptive statistics were used to summarise baseline patient characteristics and waiting times along the PCCP steps. Numerical variables, including clinic waiting times, were reported as medians with interquartile ranges (IQRs) due to their non‐normal distribution, which was inspected by visual inspection of their respective histograms. Categorical variables, such as patient pathway distributions and questionnaire response rates, were presented as frequencies and percentages.

Differences in waiting times between the three independent patient cohorts (PCCP patients, pre‐PCCP patients and COVID‐19 lockdown patients) were assessed using the Kruskal‐Wallis test, as the datasets included three independent groups and were non‐parametric.

All statistical analyses were conducted using GraphPad Prism Version 10.0.1 (218), with a significance threshold set at p < 0.05. Missing data were not imputed, and patients with incomplete data were excluded from analysis.

## 3 RESULTS

The follow‐up appointments of 432 patients were analysed in this study after their prostate biopsies. Of these, 248 patients underwent the PCCP following its implementation in July 2022, while 75 patients were assessed immediately prior to PCCP implementation and 75 during the second Victorian COVID‐19 lockdown. Thirty‐four patients were excluded, including 32 who sought further follow‐up and/or treatment outside of Monash Health and two with incomplete data. The final cohort for analysis included 398 patients.

Patients across the three groups had comparable median age and pre‐operative serum PSA, as well as a similar distribution of biopsy‐naïve patients and ISUP PCa grades (Table 1). A higher proportion of patients in the Pre‐PCCP 2020 group (49.33%) were discharged compared to Pre‐PCCP 2022 (17.33%) and Post‐PCCP 2022 (20.56%) groups. Furthermore, a comparable proportion of patients were referred for MDT discussion, Urology Consultant review and Radiation Oncology Consultant review in the three groups.

**TABLE 1:** Clinicopathological characteristics and post‐biopsy pathways of the study population.

		Post‐PCCP (n = 248)	Pre‐PCCP 2022 (n = 75)	Pre‐PCCP 2020 (n = 75)
**Median Age in years (IQR)**		69.0 (62.5–75.1)	68.6 (63.6–73.3)	69.0 (64.4–74.4)
**Patients >70 years (%)**		114 (46.0%)	28 (37.3%)	30 (40.0%)
**Patients >80 years (%)**		11 (4.4%)	2 (2.7%)	6 (8.0%)
**Median Preoperative serum PSA level in ng/mL (IQR)**		8.1 (5.6–11.3)	8.1 (5.8–12.7)	8.0 (5.5–13.0)
**Biopsy‐naïve patients (%)**		168 (67.7%)	50 (66.7%)	55 (73.3%)
**Biopsy Grading**	Benign Biopsy	76 (30.7%)	21 (28.0%)	27 (36.0%)
	ISUP 1 (Gleason 3 + 3)	48 (19.4%)	20 (26.7%)	18 (24.0%)
	ISUP 2 (Gleason 3 + 4)	56 (22.6%)	23 (30.7%)	15 (20.0%)
	ISUP 3 (Gleason 4 + 3)	29 (11.7%)	3 (4.0%)	5 (6.7%)
	ISUP 4 (Gleason 8)	10 (4.0%)	1 (1.3%)	7 (9.3%)
	ISUP 5 (Gleason 9/10)	27 (10.9%)	12 (16.0%)	3 (4.0%)
**Post‐biopsy Pathways**	Discharged	51 (20.6%)	13 (17.3%)	37 (49.3%)
	Active surveillance	67 (27.0%)	30 (40.0%)	5 (6.7%)
	MDT, Urology & Radiation Oncology Consultant review	130 (52.4%)	37 (49.3%)	33 (44.0%)

ISUP, International Society of Urological Pathology; MDT, Multidisciplinary Team.

Table 2 demonstrates that patients in the PCCP pathway had significantly shorter median wait times for post‐biopsy review clinic appointments (15 days, IQR 13–19) compared to both the pre‐PCCP 2022 cohort (21 days, IQR 17–27) and the pre‐PCCP 2020 cohort (18 days, IQR 14–21; p < 0.001). Similarly, times from biopsy to Radiation Oncology and/or Urology Consultant review were significantly reduced in the PCCP cohort (38 days, IQR 31–49) compared to the pre‐PCCP 2022 cohort (63 days, IQR 46–82) and the pre‐PCCP 2020 cohort (52 days, IQR 40–75; p < 0.001). However, there was no significant difference in waiting times from biopsy to MDT discussion across the three groups (p = 0.360).

**TABLE 2:** Waiting times for patients following prostate biopsy.

	Post‐PCCP pathway 2022 (n = 248)	Pre‐PCCP pathway 2022 (n = 75)	Pre‐PCCP pathway 2020 (n = 75)	p‐values
**Median Days from biopsy to post‐biopsy review clinic appointment (IQR)**	15 (13–19)	21 (17–27)	18 (14–21)	p < 0.001
**Median Days from biopsy to MDT (IQR)**	38 (30–42)	49 (35–59)	38 (27–61)	p = 0.360
**Median Days from biopsy to Radiation Oncology/Urology Consultant Review (IQR)**	38 (31–49)	63 (46–82)	52 (40–75))	p < 0.001

All 248 patients who underwent the PCCP were contacted to participate in the PCQ‐P questionnaire. A total of 176 patients (71.0%) provided verbal consent and completed the questionnaire. Of these, 100 patients completed only Part 1, which assessed satisfaction with waiting times for receiving biopsy results. These patients either had benign prostate biopsies or were placed on active surveillance. The remaining 76 patients, who were diagnosed with PCa and progressed to treatment, completed both Part 1 and Part 2, which additionally evaluated satisfaction with waiting times for treatment consultations. Among the 72 patients who did not participate, 23 (31.9%) declined participation, while 49 (68.1%) could not be reached after two separate contact attempts. The PCQ‐P responses are summarised in Table 3.

**TABLE 3:** ‐ Participant responses to the modified PCQ‐P.

Modified PCQ‐P satisfaction questionnaire: part 1 questions (n = 176)		
Q1) *How did you feel about the time the GP's practice/local assessment centre took to refer you to the hospital?*	Too short	0 (0%)
	**About right**	**170 (96.6%)**
	Too long	6 (3.4%)
*Q2) Were you told at the GP's practice/local assessment centre how soon you would be seen at the hospital?*	Yes	11 (6.3%)
	**No**	**165 (93.8%)**
*Q3) How did you feel about the length of time you had to wait for your first appointment at the hospital?*	Too short	0 (0%)
	**About right**	**149 (84.7%)**
	Too long	27 (1.5%)
*Q4) After the biopsy, did the doctor or nurse explain to you how long you would have to wait for your test results?*	**Yes, the explanation was clear**	**165 (93.8%)**
	Yes, but the explanation could have been clearer	5 (2.8%)
	No explanation was given	6 (3.4%)
*Q5) How long did you have to wait from your biopsy at the hospital, until you got your diagnosis?*	**Not more than 2 weeks**	**111 (63.1%)**
	More than 2 weeks and up to 4 weeks	63 (35.8%)
	More than 4 weeks and up to 6 weeks	2 (1.1%)
	More than 6 weeks	0 (0.0%)
*Q6) After the biopsy, how did you feel about the length of time you had to wait to get your diagnosis?*	**About right**	**163 (92.6%)**
	Too long	13 (7.4%)

Modified PCQ‐P Satisfaction Questionnaire: Part 2 Questions (n = 76)		
*Q7) How did you feel about the length of time between being given your diagnosis and discussing your treatment options?* (n = 76)	Too short	0 (0%)
	**About right**	**67 (88.2%)**
	Too long	9 (11.8%)
*Q8) How did you feel about the length of time you had to consider your treatment options before the treatment decision was made?* (n = 76)	Too short	2 (2.6%)
	**About right**	**65 (85.5%)**
	Too long	9 (11.8%)
*Q9) How did you feel about the length of time you had to wait for your treatment to start?* (n = 51)	Too short	0 (0%)
	**About right**	**48 (94.1%)**
	Too long	3 (5.9%)

The majority of patients (96.6%) were satisfied with waiting times from their GP practice or local assessment referral to our centre and a significant majority (93.8%) expressed satisfaction with the clarity of explanations offered by our staff regarding expected waiting times for biopsy results. A total of 111 patients (63.1%) reported waiting “No more than 2 weeks” for biopsy results, which accurately correlates with patients' actual median waiting time of 15 days (IQR 13–19 days, Table 1).

For patients who had received diagnoses of PCa and progressed to treatment, a significant majority (88.2%) were satisfied with the time between receiving their diagnosis and discussing treatment, with 85.5% also being satisfied with the time to deliberate their treatment options. Of 51 patients who had started treatment, 94.1% were satisfied with the waiting time for treatment to commence.

## 4 DISCUSSION

The journey from prostate biopsy and diagnosis to treatment is often lengthy and distressing for patients and their families. The COVID‐19 pandemic further exacerbated pre‐existing delays in prostate cancer (PCa) care due to limited availability of biopsies and consultations.^13^ Prior to the pandemic, a study by Qu et al. identified median delays of 1.9 months for both prostate biopsy and treatment discussion, highlighting that care delays were a systemic issue rather than solely a result of pandemic‐related disruptions.^2^ Our study demonstrates that the implementation of the PCCP significantly reduced waiting times between biopsy and result disclosure, as well as between result disclosure and multidisciplinary treatment discussions. Notably, these improvements were achieved without requiring additional resources but rather through restructuring of existing staffing and clinic schedules. This indicates that similar pathways could be adapted in other healthcare settings to improve efficiency and patient outcomes.

Previous studies have shown mixed findings regarding the clinical significance of reducing waiting times within PCa care pathways; however, there is strong evidence that shorter waiting times improve patient satisfaction.^14^, ^15^, ^16^ Given the psychological burden of a PCa diagnosis, reducing the time spent awaiting biopsy results and treatment decisions is particularly beneficial.^17^, ^18^ Anxiety levels are highest in patients who have undergone a biopsy but have not yet received results, primarily due to uncertainty and decision‐making stress.^19^ Our findings reinforce that reducing waiting periods positively impacts patient well‐being, as demonstrated by the high satisfaction rates reported in our patient questionnaires. The majority of participants (92.6%) felt that the waiting time for biopsy results was “about right,” and 88.2% were satisfied with the waiting time between biopsy and treatment discussion. Furthermore, 93.8% of patients reported satisfaction with the clarity of expectations regarding result disclosure timelines. These outcomes highlight the importance of structured communication and collaboration between medical, nursing and administrative staff to ensure timely referrals for patient engagement and satisfaction.

Despite the substantial improvements achieved through the PCCP, further refinements could enhance its efficiency. While waiting times between biopsy and result disclosure were reduced to the targeted two‐week timeframe, the interval between biopsy and multidisciplinary team (MDT) discussion, as well as subsequent consultant review, remains an area for improvement. Efforts were made to coordinate same‐day MDT meetings and consultant reviews for high‐risk patients, but Radiation Oncology appointments were not always synchronised. The Odette Cancer Centre in Toronto successfully reduced waiting times by implementing a Rapid Diagnostic Unit (RDU), where Urology and Radiation Oncology specialists reviewed patients on the same day post‐biopsy.^20^ Adopting a similar model could help further optimise care for high‐risk PCa patients. Reducing waiting times for treatment consultations would be especially beneficial for those with symptomatic or extensive metastatic disease, ensuring alignment with guidelines that recommend initiating ADT or other systemic therapy within four weeks of diagnosis.^8^

Future PCCPs may benefit from changes to clinic structures and the collaboration between Urologists, Surgical Residents, Specialist Prostate Cancer Nurses and Administrative Staff. Such changes could include the establishment of a dedicated PCa‐specific clinic for post‐biopsy reviews and treatment planning. In a tertiary Urology centre, this structured approach could allow for greater involvement of Surgical Residents and Nurses, following standardised pathways while seeking consultant input when necessary. Such a model has the potential to enhance clinic efficiency, increase the number of patients reviewed and alleviate pressure on broader general Urology clinic services. Furthermore, models such as the One Stop Prostate Clinic (OSPC) in Western Australia emphasise multidisciplinary collaboration, including Nurse‐led telephone result notifications for rural patients with benign histopathology reports.^21^ This method of safely disclosing results for benign biopsies can allow for no further in‐person clinic appointments and have significant cost savings for the public healthcare system.^22^ Implementing a similar approach could optimise clinic time by discharging patients with benign findings to primary care, thereby reserving specialist consultations for those requiring further intervention and patients with malignant pathologies. Further investigation into Nurse‐led clinics and their role in reducing PCa treatment planning delays may provide valuable insights into optimising resource allocation and clinic efficiency, which may further help to reduce waiting times for PCa treatment planning and non‐PCa Urological clinic waiting times.

Although best efforts were made to ensure valid findings, some factors may limit result interpretation. Firstly, our data is obtained from a single tertiary centre which may limit the generalisability of our findings to other healthcare systems with different patient demographics, resource availability or institutional workflows. Furthermore, while our pre‐PCCP data collection included 150 patients during either Covid‐19 lockdowns or immediately prior to the pathway's implementation, the post‐PCCP cohort encompassed a full year of public patient follow‐up, potentially introducing seasonal variation or incomplete data biases when comparing to Pre‐PCCP cohorts. Additionally, patient satisfaction was assessed using the Modified PCQ‐P only for those who underwent the PCCP, which inadvertently limits direct comparisons with pre‐PCCP cohorts. The questionnaire was specifically addressed at only patients who had undergone the PCCP and excluded pre‐PCCP patients to minimise recall bias. We were also unable to collect additional variables that may have influenced our findings, including missed or cancelled appointments, private specialist appointments, patients' ethnicities or geographical distances from the clinic. Despite these limitations, our study provides valuable insight into the experiences of three patient cohorts before and after the implementation of a structured prostate cancer care pathway. Our findings underscore the impact of effective risk stratification and multidisciplinary collaboration in reducing patient waiting times while maintaining high levels of patient satisfaction.

Future interventions should focus on reducing waiting times between biopsy, MDT discussion and treatment planning. Potential strategies include same‐day Urology and Radiation Oncology reviews within a dedicated PCa clinic and expanding Nurse/Resident‐led consultations under PCCP guidelines with consultant oversight. These innovations have the potential to enhance patient care while improving overall Urology service efficiency. By continuing to refine and optimise the PCCP, healthcare systems can better address delays in PCa diagnosis and treatment, ultimately improving patient outcomes and satisfaction.

## 5 CONCLUSION

The implementation of the PCCP significantly reduced waiting times for biopsy result disclosure and treatment discussions, demonstrating that restructuring existing resources can enhance efficiency in a large, tertiary centre. High patient satisfaction rates further highlight the benefits of streamlined, multidisciplinary collaboration in prostate cancer care. Future efforts should focus on optimising MDT discussions and treatment planning through dedicated PCa clinics and expanded Nurse/Resident‐led consultations to further improve patient outcomes and healthcare service efficiency.

## CONFLICT OF INTEREST STATEMENT

None of the authors have any conflicts of interest to declare.

## Supporting information


**Data S1.** Supporting Information

## References

[1] Bray F , Laversanne M , Sung H , Ferlay J , Siegel RL , Soerjomataram I , et al. Global cancer statistics 2022: GLOBOCAN estimates of incidence and mortality worldwide for 36 cancers in 185 countries. CA Cancer J Clin. 2024 May;74(3):229–263. 10.3322/caac.21834 38572751

[2] Qu LG , Nzenza T , McMillan K , Sengupta S . Delays in prostate cancer care within a hospital network in Victoria, Australia. ANZ J Surg. 2019;89:1599–1604. 31786815 10.1111/ans.15554

[3] Valbekmo AG , Mo L , Gjøsund G , Håland E , Melby L . Exploring wait time variations in a prostate cancer patient pathway—a qualitative study. Int J Health Plann Manage. 2022;37(4):2122–2134. 10.1002/hpm.3454 35347768 PMC9543572

[4] Robertson S , Adolfsson J , Stattin P , Sjövall A , Winnersjö R , Hanning M , et al. Waiting times for cancer patients in Sweden: a nationwide population‐based study. Scand J Public Health. 2017;45(3):230–237. 10.1177/1403494817693695 28443490

[5] van Hoeve JC , Vernooij RWM , Fiander M , Nieboer P , Siesling S , Rotter T . Effects of oncological care pathways in primary and secondary care on patient, professional and health systems outcomes: a systematic review and meta‐analysis. Syst Rev. 2020;9(1). 10.1186/s13643-020-01498-0 PMC7586678 33100227

[6] García‐Rojo E , Manfredi C , Santos‐Pérez‐de‐la‐Blanca R , Tejido‐Sánchez Á , García‐Gómez B , Aliaga‐Benítez M , et al. Impacto del brote de COVID‐19 en las listas de espera de cirugía urológica y estrategias de priorización en la era post‐COVID‐19. Actas Urol Esp. 2021;45(3):207–214. 10.1016/j.acuro.2020.11.001 33546905

[7] Kizilkan Y , Senel S , Ozercan AY , Balci M , Eroglu U , Aktas BK , et al. Evaluating the anxiety and depression status of prostate cancer patients whose operations were postponed because of the COVID‐19 pandemic. Int J Clin Pract. 2021;75(8):e14278. 10.1111/ijcp.14278 33914983 PMC8236926

[8] Thomas RJS . Optimal care pathway for men with prostate cancer [Internet]. 2021. Available from: https://www.racgp

[9] NHS Improvement ‐ Cancer . Stratified pathways of care… from concept to innovation Executive Summary [Internet]. 2012. Available from: www.improvement.nhs.uk/cancer/survivorship

[10] Sinfield P . University of Leicester. 2011. Developing a measure of patient experience of prostate cancer care.

[11] Tarrant C , Baker R , Colman AM , Sinfield P , Agarwal S , Mellon JK , et al. The prostate care questionnaire for patients (PCQ‐P): reliability, validity and acceptability. BMC Health Serv Res. 2009 Nov 4;;9(199). 10.1186/1472-6963-9-199 PMC2777154 19889223

[12] Prostate Cancer Foundation of Australia . Prostate cancer ‐ A guide for newly‐diagnosed men. 2024.

[13] Nossiter J , Morris M , Parry MG , Sujenthiran A , Cathcart P , van der Meulen J , et al. Impact of the COVID‐19 pandemic on the diagnosis and treatment of men with prostate cancer. BJU Int. 2022;130(2):262–270. 10.1111/bju.15699 35080142

[14] Osowiecka K , Nawrocki S , Kurowicki M , Rucinska M . The waiting time of prostate cancer patients in Poland. Int J Environ Res Public Health. 2019;16(3):342. 10.3390/ijerph16030342 30691113 PMC6388381

[15] Lazzeri G , Troiano G , Porchia BR , Centauri F , Mezzatesta V , Presicce G , et al. Waiting times for prostate cancer: a review. J Public Health Res. 2020;9(1):1778. 10.4081/jphr.2020.1778 32550222 PMC7282316

[16] Gacci M , Greco I , Artibani W , Bassi P , Bertoni F , Bracarda S , et al. The waiting time for prostate cancer treatment in Italy: analysis from the PROS‐IT CNR study. Minerva Urol Nephrol. 2022 Feb;74(1):38–48. 10.23736/S2724-6051.20.03925-9 33200896

[17] Braga R , Costa AR , Pina F , Moura‐Ferreira P , Lunet N . Prostate cancer screening in Portugal: prevalence and perception of potential benefits and adverse effects. Eur J Cancer Prev. 2020 May;29(3):248–251. 10.1097/CEJ.0000000000000539 31651568

[18] Watts S , Leydon G , Birch B , Prescott P , Lai L , Eardley S , et al. Depression and anxiety in prostate cancer: a systematic review and meta‐analysis of prevalence rates. BMJ Open. 2014 Mar;4(3):e003901. 10.1136/bmjopen-2013-003901 PMC3963074 24625637

[19] Gray RE , Fitch MI , Phillips C , Labrecque M , Klotz L . Presurgery experiences of prostate cancer patients and their spouses. Cancer Pract. 1999 May;7(3):130–135. 10.1046/j.1523-5394.1999.07308.x 10352075

[20] Sethukavalan P , Zhang L , Jethava V , Stevens C , Flax S , Buckley R , et al. Improved wait time intervals for prostate cancer patients in a multi‐disciplinary rapid diagnostic unit compared to a community‐based referral pattern. Can Urol Assoc J. 2013;7(7–8):244–250. 10.5489/cuaj.181 24032058 PMC3758939

[21] McCombie SP , Hawks C , Emery JD , Hayne D . A ‘one stop’ prostate clinic for rural and remote men: a report on the first 200 patients. BJU Int. 2015;116(S3):11–17. 10.1111/bju.13100 26218767

[22] Hawks C , Al‐Zubaidi M , Viswambaram P , Gonsalves J , Brown M , Byrnes J , et al. Analysis of the financial impact and efficiency of the one stop prostate clinic: a same day prostate cancer diagnostic clinic in the Australian public health system. J Public Health Res. 2023;12(1):227990362211468. 10.1177/22799036221146882 PMC9834939 36643606

