# Supplementary material for: Optimising prostate cancer pathways: Improving post‐biopsy waiting times in a tertiary centre
Source: BJUI Compass. 2025 Jul 2;6(7):e70045. doi: 10.1002/bco2.70045 (PMC12221808; doi:10.1002/bco2.70045)
Supplement: Supplementary file 1 — Data S1. Supporting Information [file BCO2-6-e70045-s001.docx]

Modified Prostate Cancer Questionnaire for Patients (PCQ-P)

1. How did you feel about the time the GP’s practice/local assessment centre took to refer you to the hospital?
   1. Too short
   2. About right
   3. Too long
2. Were you told at the GP’s practice/local assessment centre how soon you would be seen at the hospital?
   1. No
   2. Yes
3. How did you feel about the length of time you had to wait for your first appointment at the hospital?
   1. Too short
   2. About right
   3. Too long
4. After the biopsy, did the doctor or nurse explain to you how long you would have to wait for your test results?
   1. Yes, the explanation was clear
   2. Yes, but the explanation could have been clearer
   3. No explanation was given
5. How long did you have to wait from your biopsy at the hospital, until you got your diagnosis?
   1. Not more than 2 weeks
   2. More than 2 weeks and up to 4 weeks
   3. More than 4 weeks and up to 6 weeks
   4. More than 6 weeks
6. After the biopsy, how did you feel about the length of time you had to wait to get your diagnosis?
   1. About right
   2. Too long

Ask the following only if the patient was diagnosed with Prostate Cancer.

1. How did you feel about the length of time between being given your diagnosis and discussing your treatment options?
   1. Too short
   2. About right
   3. Too long
2. How did you feel about the length of time you had to consider your treatment options before the treatment decision was made?
   1. Too short
   2. About right
   3. Too long
3. How did you feel about the length of time you had to wait for your treatment to start?
   1. Too short
   2. About right
   3. Too long
